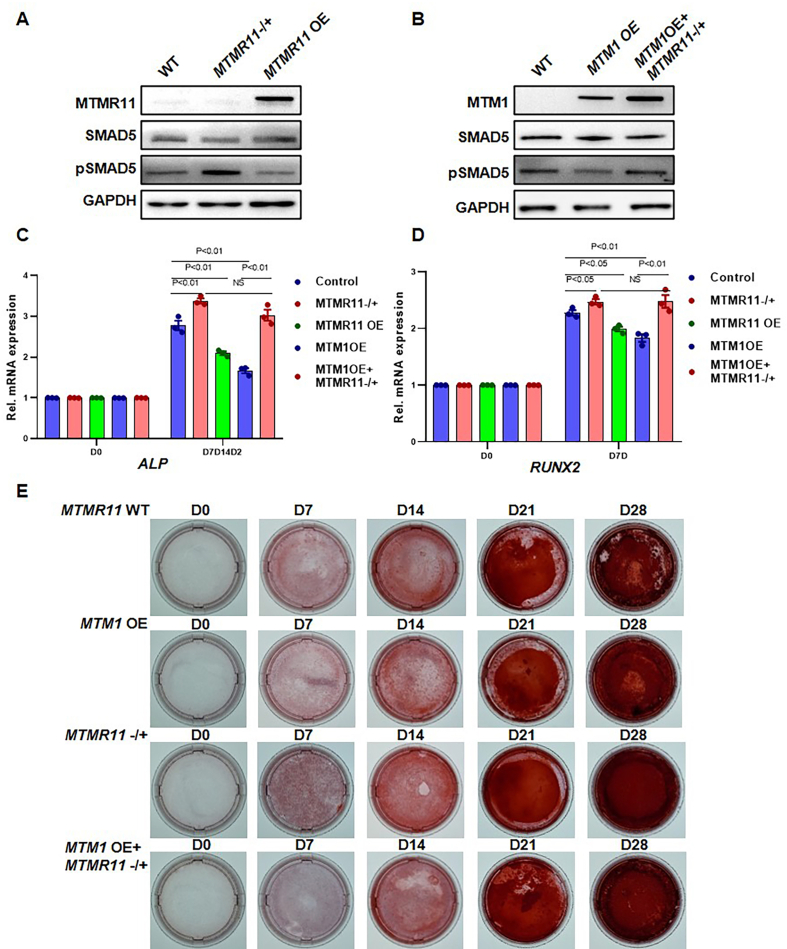# The *MTMR11* variants identified in a short stature cohort compromise the dephosphorylation ability of *MTM1* on SMAD5 to up-regulate BMP signaling

**DOI:** 10.1016/j.gendis.2024.101393

**Published:** 2024-08-21

**Authors:** Kai Yang, Hongdou Li, Rui Peng, Bo Wu, Yiping Shen, Tongjin Zhao, Chentao Li, Weimin Wang, Hongyan Wang

**Affiliations:** a Obstetrics & Gynecology Hospital, State Key Laboratory of Genetic Engineering, School of Life Sciences, Fudan University, Shanghai 200032, China; b Prenatal Diagnosis Center of Shenzhen Maternity & Child Healthcare Hospital, Shenzhen, Guangdong 518028, China; c Genetic and Metabolic Central Laboratory, Birth Defects Prevention and Control Institute of Guangxi Zhuang Autonomous Region, Maternal and Child Health Hospital of Guangxi Zhuang Autonomous Region, Nanning, Guangxi 530003, China; d Shanghai Key Laboratory of Metabolic Remodeling and Health, Institute of Metabolism and Integrative Biology, Fudan University, Shanghai 200438, China; e Shanghai Medical College, Fudan University, Shanghai 200032, China; f Department of Pharmacy College of Life Sciences, China Jiliang University, Hangzhou, Zhejiang 310018, China; g Children's Hospital, Fudan University, Shanghai 201102, China

Short stature is clinically defined as a standing height less than two standard deviations below the mean height at the same age, ethnicity, and sex. As a typical complex symptom, height has a high heritability of 80%, which is affected by multiple genes and gene–gene interactions. A genome-wide association study (GWAS) revealed that 23.3% of the heritability of short stature could be explained by 697 independent variants.^1^ Whole exome sequencing of a large sample size demonstrated that 83 rare and low-frequency variants could explain 1.7% heritability of height, and variants with minor allele frequency <5% have an average effect 10 times greater than that of common variants.^2^ Here, we analyzed the whole exome sequencing data of 787 short-stature children to find new genes contributing to short stature in Chinese children.

It is well known that excessive differentiation of osteoblasts leads to short stature, and the normal differentiation of osteoblasts is regulated by bone morphogenetic protein (BMP) signaling.^3^ As a core regulatory member, phosphorylated SMAD family member 5 (pSMAD5) is positively correlated with BMP signaling activity, but it is unclear how SMAD5 dephosphorylation is regulated during osteoblast development. Myotubularin-related protein 11 (MTMR11), which belongs to the myotubularin family, was predicted to be a dead phosphatase due to the absence of conserved cysteine residues in catalytic activity domains and loss of its phosphatase activity. To date, no data support *MTMR11* as a disease-causing gene, even though the connection of myotubularin 1 (MTM1) with short stature is also unclear^4^ until we found that rare MTMR11 variants were enriched in our dwarfism cohort.

We identified *MTMR11* heterozygous variants from short stature (Fig. S1), and found overexpression (OE) of *MTMR11* inhibited the BMP signaling pathway (Fig. S2A), but knockout (KO) of *MTMR11* displayed higher expression of the BMP pathway marker genes collagen type X alpha 1 chain (*COL10A1*), sclerostin (*SOST*), matrix metallopeptidase 13 (*MMP13*), and RUNX family transcription factor 2 (*RUNX2*) (Fig. S2F). Coimmunoprecipitation assays showed that MTMR11 could interact with SMAD5 in the HEK293T cell line (Fig. S2G). We next are curious about who can interact with MTMR11 to inhibit the BMP signaling pathway. Mass spectrometry analysis showed an interaction between MTM1 and MTMR11. Luciferase assay showed the inhibitory role of *MTM1* on the BMP signaling pathway, which was further exaggerated by cotransfection with *MTMR11* (Fig. S3A, D). Also, the 3-PAP domain was identified be the core domain for the interaction between MTM1 and MTMR11 (Fig. S3C). On the way to figure out how MTM1 and MTMR11 inhibit the BMP pathway, we found that MTMR11 could band to SMAD5, which is the core factor in the BMP pathway. Then we found *MTMR11* acted as an essential co-factor to enhance MTM1 dephosphorylation of pSMAD5 (Fig. S4).

The IDG-SW3 cell line was a helpful tool for osteoblast study, whose differentiation was induced by removing interferon-gamma (IFN-γ; Invitrogen) and adding 50 μg/mL ascorbic acid and 4 mM β-glycerophosphate. We found *MTMR11* accelerated proliferation and inhibited osteoblast differentiation of IDG-SW3 cells by constructing the MTMR11 OE/KO cell line (Fig. S5). However, MTM1's inhibition of osteoblast differentiation in IDG-SW3 cells was compromised by knockout of *MTMR11* (Fig. S6).

To sum up, MTMR11 interacts with MTM1 to enhance SMAD5 dephosphorylation and thus inhibit the BMP pathway and maintain the balance of osteoblast differentiation. The MTMR11 variants identified in a short stature cohort compromise SMAD5 dephosphorylation for abnormally up-regulated BMP signaling, thus causing short stature.

The process of osteogenesis is finely regulated by BMP signals, which are crucial to regulating the cell proliferation and differentiation of osteoblasts into osteocytes. In this study, we identified four rare variants of *MTMR11*, a dead phosphatase gene, in a cohort of 787 short-stature children. Mechanistically, MTMR11 can interact with both SMAD5 and MTM1 and work as a necessary cofactor for the active phosphatase MTM1 to specifically work on SMAD5 protein dephosphorylation and to inhibit BMP signaling, and this interaction is significant for the balance of osteoblast development. The two short stature-enriched *MTMR11* variants c.1225C > T (p. R409X) and c.1780C > T (p. R594X) destroyed the 3-PAP domain and lost the ability to interact with MTM1, which led to the interruption of the regular inhibition of BMP signaling. The MTMR11 variants induced overactivated BMP signaling through pSMAD5 accumulation, subsequently promoting the over-differentiation of osteoblasts, which contributed to short stature in carriers and conferred *MTMR11* as a short stature likely pathogenic gene (Fig. 1).

**Figure 1:**
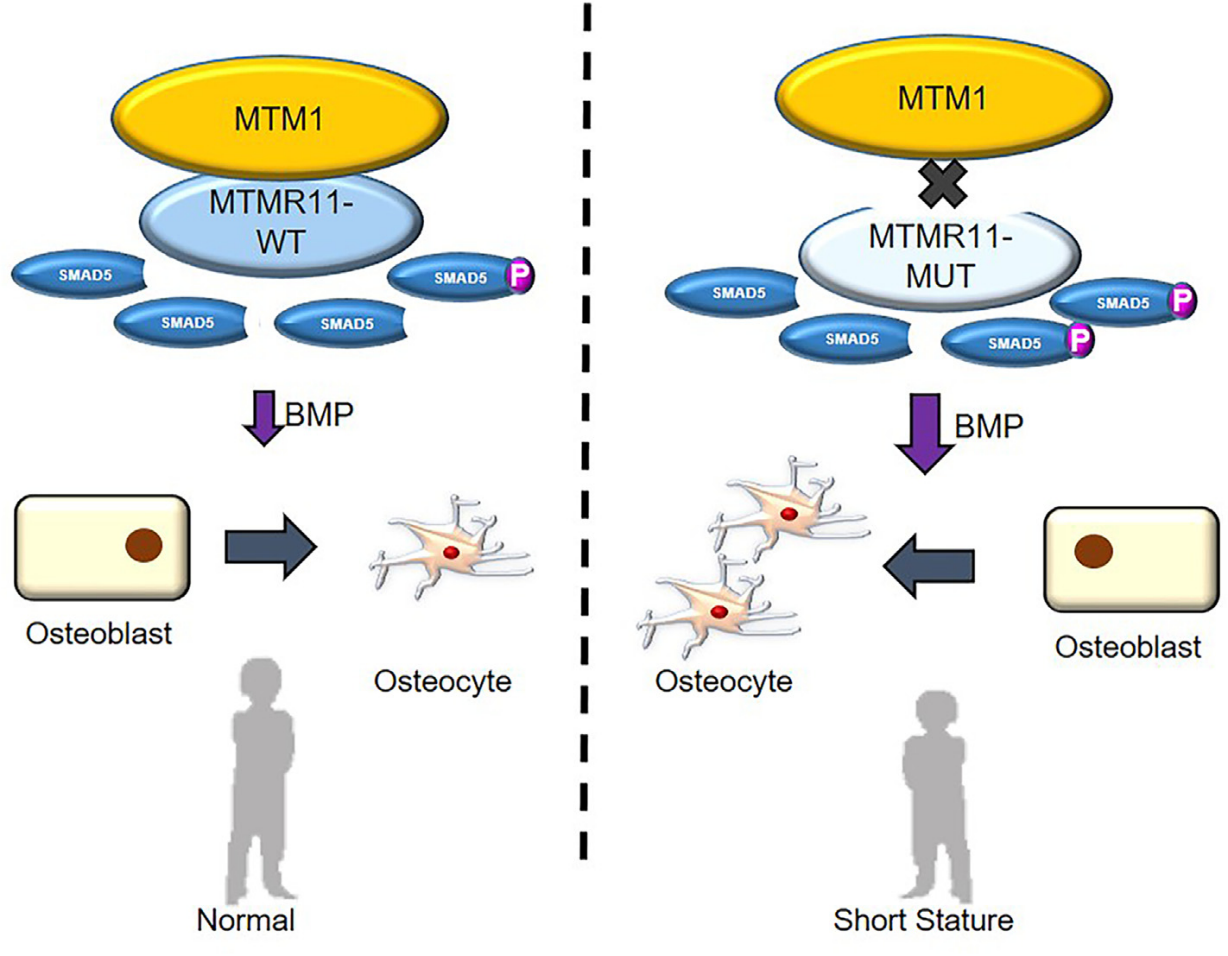
Abstract graphic of functional MTMR11 in short stature development.

After we revealed the possible pathogenic role of MTMR11, a dead phosphatase, to short stature, MTM1 was further demonstrated to be the real functional phosphatase for the substrate of protein SMAD5 because it was reported that dead phosphatase members could form a dimer with members in the same family containing active phosphatase domains to enhance or inhibit their phosphatase activity. Clearly, the irreplaceable importance of MTM1 as a protein phosphatase was linked to the finding of a connection between *MTMR11* and short stature. However, it remains ambiguous whether cofactors such as MTMR11 are necessary only for the protein phosphatase function of MTM1 or whether a certain cofactor is also needed for MTM1 to execute lipid phosphatase activity. Collectively, our study clarified the correlation between the regulation of MTM1 protein phosphatase activity and dwarfism occurrence.

Our definition of MTM1 as a new phosphatase for the SMAD5 protein will helpfully elucidate the sophisticated regulation of the BMP pathway and inspire more attention to protein phosphatase studies. Therefore, our findings elucidated a new dephosphorylation pathway for SMAD5 regulation that causes imbalanced BMP signal activity and subsequent short stature outcomes, which provided different insights concerning the pathogenic factors for dwarfism. Hopefully, new protein phosphatases or cofactors in the BMP signaling pathway could be potential drug targets for skeletal development and short stature therapy.

## Ethics declaration

The project was approved by the Institutional Medical Ethics Review Board of the Maternal and Child Health Hospital of Guangxi Zhuang Autonomous Region (No. (2015)-4-16]. Informed consent was obtained from the parents and the patients.

## Conflict of interests

There is no conflict of interest.

## Funding

This work was supported by the National Key R&D Program of China (No. 2021YFC2701100 to H.W.), the National Natural Science Foundation of China (No. 81930036, 8215008 to H.W.), and the Commission for Science and Technology of Shanghai Municipality, China (No. 20JC1418500 to H.W.).

## CRediT authorship contribution statement

**Kai Yang:** Data curation, Formal analysis, Validation, Visualization, Writing – original draft, Writing – review & editing. **Hongdou Li:** Writing – review & editing. **Rui Peng:** Writing – review & editing. **Yiping Shen:** Data curation, Investigation. **Tongjin Zhao:** Data curation. **Chentao Li:** Data curation. **Weimin Wang:** Writing – original draft, Writing – review & editing. **Hongyan Wang:** Project administration, Writing – original draft, Writing – review & editing.

## Figures and Tables

**figs1:**
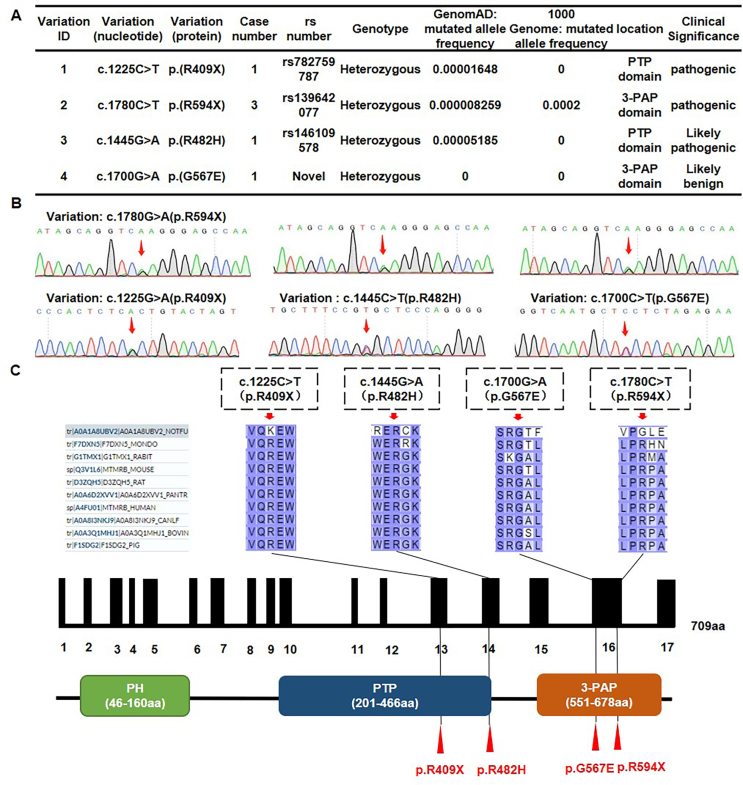


**figs2:**
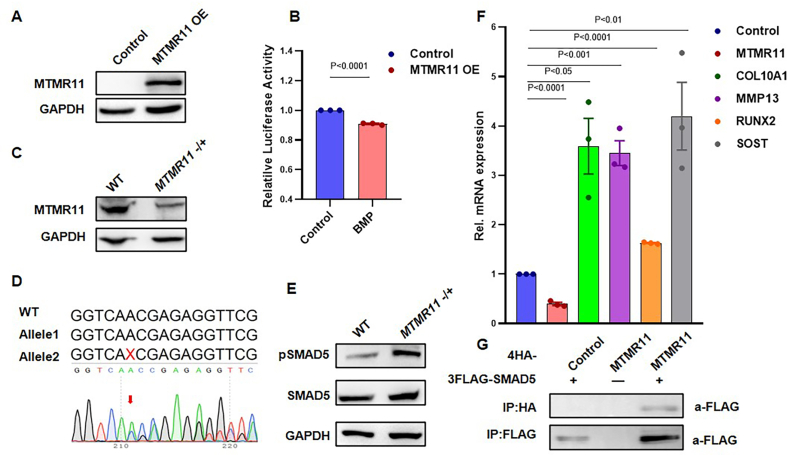


**figs3:**
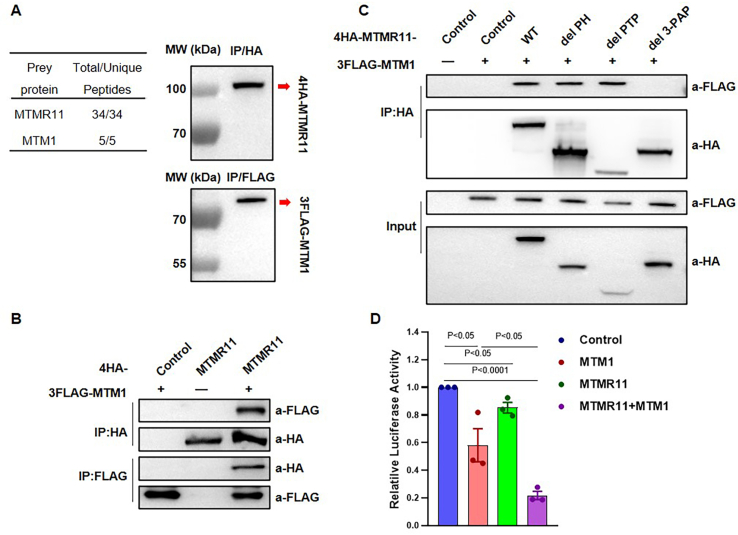


**figs4:**
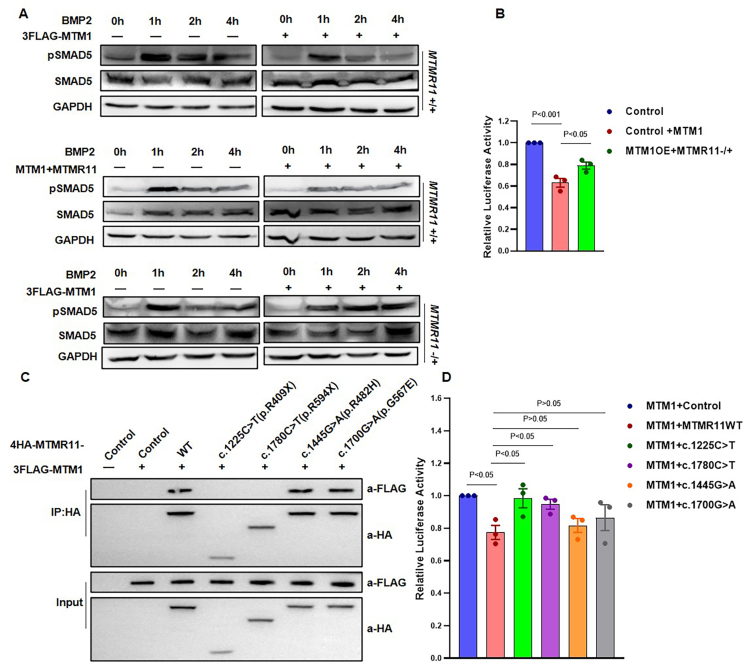


**figs5:**
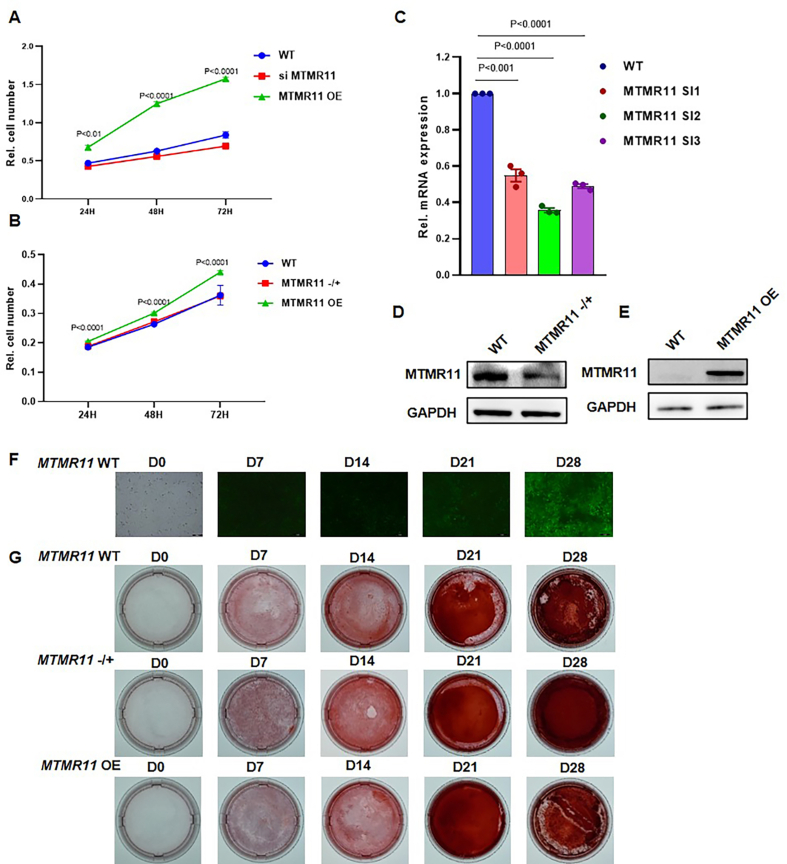


**figs6:**