# Supplementary material for: The MTMR11 variants identified in a short stature cohort compromise the dephosphorylation ability of MTM1 on SMAD5 to up-regulate BMP signaling
Source: Genes Dis. 2024 Aug 21;12(4):101393. doi: 10.1016/j.gendis.2024.101393 (PMC11999603; doi:10.1016/j.gendis.2024.101393)
Supplement: Multimedia component 1 [file mmc1.docx]

**Materials and methods**

**Patients**

Our short stature cohort was composed of 787 short stature patients recruited from 25 hospitals in China. All patients were referred to pediatric endocrinologists for clinical evaluation of short stature (< 2.5 SD). Patients with other congenital malformations or systemic disorders with secondary eﬀects on growth were excluded from the cohort. They all participated in a multicenter short stature sequencing program organized by the Maternal and Child Health Hospital of Guangxi Zhuang Autonomous Region. The project was approved by the Institutional Medical Ethics Committee. Clinical data and biological material of 5 ml peripheral blood samples were collected from the participating family after written informed consent.

**DNA extraction and next-generation sequencing**

Genomic DNA was extracted from peripheral blood samples obtained from patients and available parents using the GentraPuregene Blood Kit (QIAGEN, Hilden, Germany) according to the manufacturer's protocol. Next-generation sequencing was performed by either HiSeq 2000 or HiSeq X Ten (Illumina, San Diego, CA, USA). Paired sequences obtained from each sample were aligned to the GRCh37/hg19 human reference sequence using Burrows‒Wheeler Aligner (BWA) with the MEM algorithm. Variants were annotated by the Genome Analysis Toolkit (GATK).

**Evaluation and validation of genetic variants**

Population frequencies of the detected variants were assessed using gnomAD (https://gnomad.broadinstitute.org/), 1000 Genomes data (http://www.internationalgenome.org), and 592 Chinese normal-height controls. Clinical significance was tested by Polyphen-2. Candidate pathogenic variants were confirmed by Sanger sequencing.

**Construction of the *MTMR11* variants Plasmids**

The full-length human *MTMR11* complementary DNA was amplified from the cDNA of the U-2OS cell line, cloned and inserted into the 4HA-PCS2 vector by the restriction enzyme cutting site MluⅠ, NheⅠ and Phusion High-Fidelity DNA Polymerase (NEB) to generate the MTMR11 expression plasmid. Specific primers were designed based on the *MTMR11* sequence (ENST00000439741.2) in ensemble (http://asia.ensembl.org/Homo_sapiens/Info/Index). *MTMR11* site-directed variants were generated by overlap PCR with KOD Neo Plus Polymerase (TOYOBO). All cloned sequences were verified by Sanger sequencing.

**Cell culture and cell Differentiation**

The human osteosarcoma cell Line U-2OS and kidney cell line HEK293T were cultured in DMEM (High Glucose, Gibco) with 10% fetal bovine serum (FBS, Gibco) and 100 units/ml penicillin‒streptomycin (Gibco) at 37 °C under 5% CO2.

The immortal mouse long bone cell line (IDG-SW3) was maintained in αMEM (Gibco) with 10% fetal bovine serum (Gibco), 100 units/ml penicillin‒streptomycin (Gibco), and 25 units/ml IFN-γ (Invitrogen) at 33 °C under 5% CO2. Before use, the culture dishes were coated with 0.15 mg/ml rat tail type I collagen (Thermo).

Differentiation of the IDG-SW3 cell line was induced by removing IFN-γ (Invitrogen), adding 50 µg/ml ascorbic acid and 4 mM β-glycerophosphate, and culturing at 37 °C under 5% CO2. The differentiation medium was refreshed every 2~3 days. The first day that the differentiation medium was changed was defined as Day 0. The cells expressed GFP under the control of the Dmp1 promoter, which was initially observed at Days 3~4 and gradually enhanced until the maximum was reached at Days 10~14.

**Transient transfection and Dual-Luciferase® Reporter Assay（Promega）**

HEK293T cells were transfected with plasmid using Lipofectamine 2000 (Invitrogen) according to the manufacturer's instructions. U-2OS and IDG-SW3 cells were transfected using GP-transfect-Mate (GenePharma). The pGL3-BRE vector and 4HA-MTMR11-PCS2 vector were cotransfected into the cell line HEK293T. The detailed procedure was performed according to the instructions of the Dual-Luciferase® Reporter Assay System. Cells were harvested, and luciferase activities were measured using a Glomax 96 microplate luminometer (Promega) after 48 hours of transfection. Renilla was used as the internal control.

**Quantitative RT‐PCR analysis**

Total RNA from the cell lines HEK293T, U-2OS, and IDG-SW3 was extracted with TRIzol reagent (TIANGEN). The RNA was reverse-transcribed into complementary cDNA using the FastQμant RT Kit (TIANGEN). Real-time PCR was performed using Universal SYBR qPCR Master Mix and QuantStudio (Applied Biosystems). GAPDH was used as the internal control.

**Western Blotting for Detection of MTMR11 and Phosphorylated-SMAD5**

All *MTMR11* variants and WT were transfected into HEK293T cells to determine the expression level of *MTMR11* variants by Western blotting. Cells were collected using cell lysis buffer for Western blotting and IP (Beyotime) after 48 hours of transfection. The primary rabbit anti-SMAD5 (CST, 1:1000 dilution) and MTMR11 (Affinity) antibodies were incubated at 4 °C overnight. After 20 minutes of TBST washing three times, the secondary antibody (goat anti-mouse IgG HRP, Abmart, 1:10000 dilution) was added and incubated for 2 hours at room temperature. Detection of MTMR11 and SMAD5 signals was visualized using a Tanon 5200 Chemiluminescence imager and Ultra High Sensitivity ECL Kit (Vazyme). All immunoblot data shown here were obtained from at least three independent experiments.

**Detection of cell proliferation by CCK8**

Ninety-six -well plates were prepared in advance, preheated 100 μL medium was added to each well. Five thousand cells were dispersed in each well and incubated for 24 hours. Ten microliters of CCK-8 reagent were added to every well for 2 hours, and then the absorbance was measured at 450 nm every 24 hours for 72 hours.

**Alizarin red S staining**

Cells were plated in collagen-coated plates and induced to differentiate on Days 0/7/14/21/28. Then, the cells were washed three times with phosphate-buffered saline (PBS), fixed with 1~2 mL 4% formaldehyde for 30 min, 4% formaldehyde was replaced with 1 mL PBS, and the cells were stained by adding 1 mL Alizarin red S for 5 min at room temperature. After staining, the cells were washed with distilled water three times.

**Construction of *MTMR11* KO and MTMR11-overexpressing cell lines**

Guide RNA was designed to target human *MTMR11* and mouse *MTMR11* exon 2. The PFUGW plasmid with guide RNA was transfected into HEK293T cells. After 12 hours of transfection, the medium was refreshed, and sodium butyrate was added to a concentration of 10 mmol. After 48 hours of transfection, a 0.45 μM filter was used to purify the supernatant to obtain lentivirus. HEK293T and IDG-SW3 cells were plated, and medium with an equal volume of lentivirus was added. Flow cytometry was used to sort cells with green fluorescence markers. All cell lines were verified by Sanger sequencing, RT‒qPCR and western blot.

**Statistical Analyses**

The differences between groups were examined by the two-tailed t test. The data are presented as the mean ± SD of at least three independent experiments. Differences were considered significant if P < 0.05, P < 0.01, and P < 0.001.


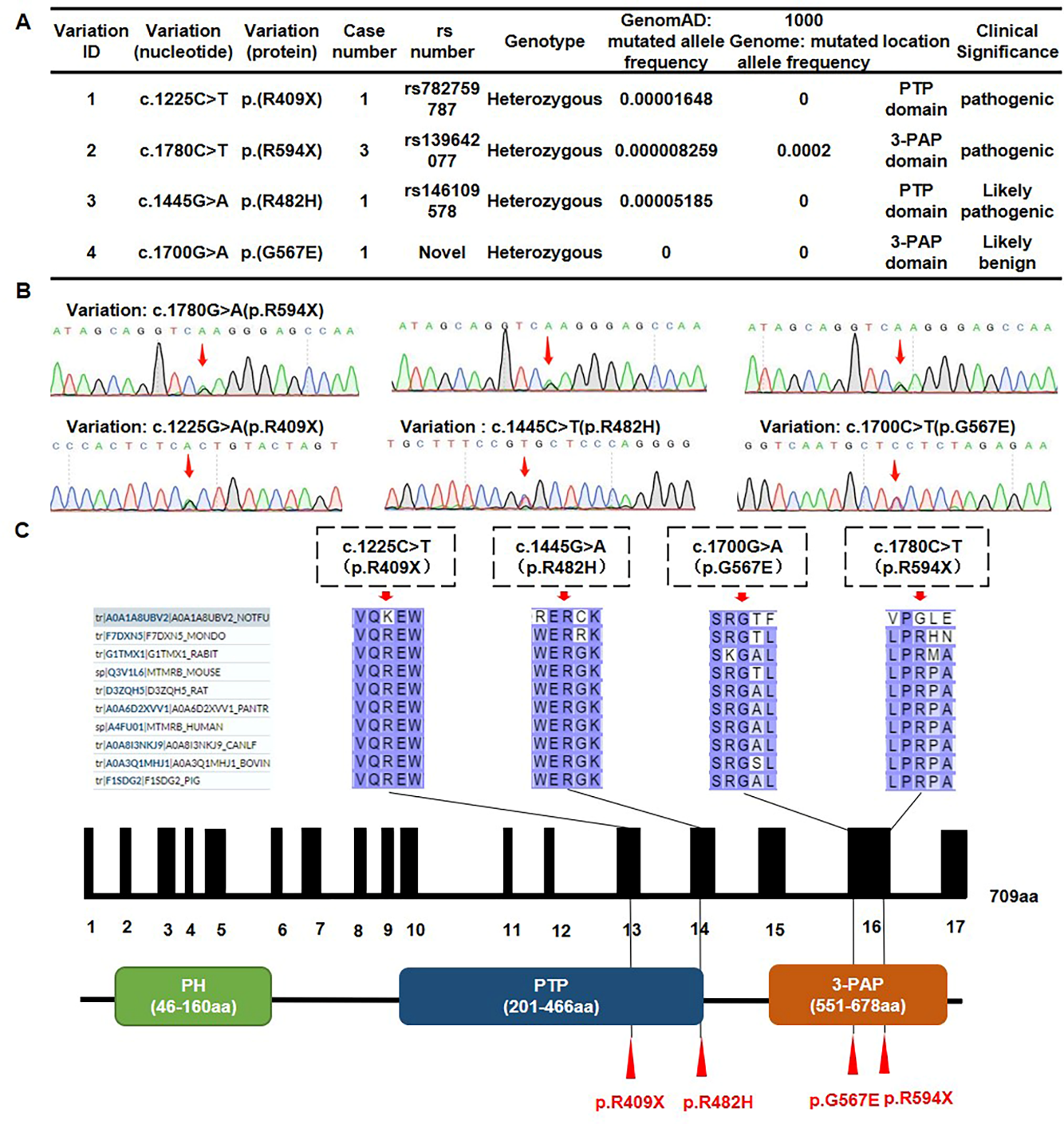


**Fig. S1 Schematic representation of the 4 identified *MTMR11* heterozygous variants*.*** (A) Each variant frequency was less than 0.0052% in the gnomeAD dataset; two stop-gain variants, R409X and R594X, were predicted by ACMG-AMP classification as pathogenic variants, while variant R482H was likely pathogenic, and variant G567E was likely benign. (B) The four reported variants identified in 6 short stature cases were validated by Sanger sequencing results. (C) Alignment shows the conservation and locations of identified *MTMR11* variants. The black boxes represent 17 exons of *MTMR11*. The colored boxes represent the three domains of MTMR11, including the PH domain (green), PTP domain (blue), and 3-PAP domain (orange). Red arrows and triangles represent the locations of variants in domains of the MTMR11 protein.


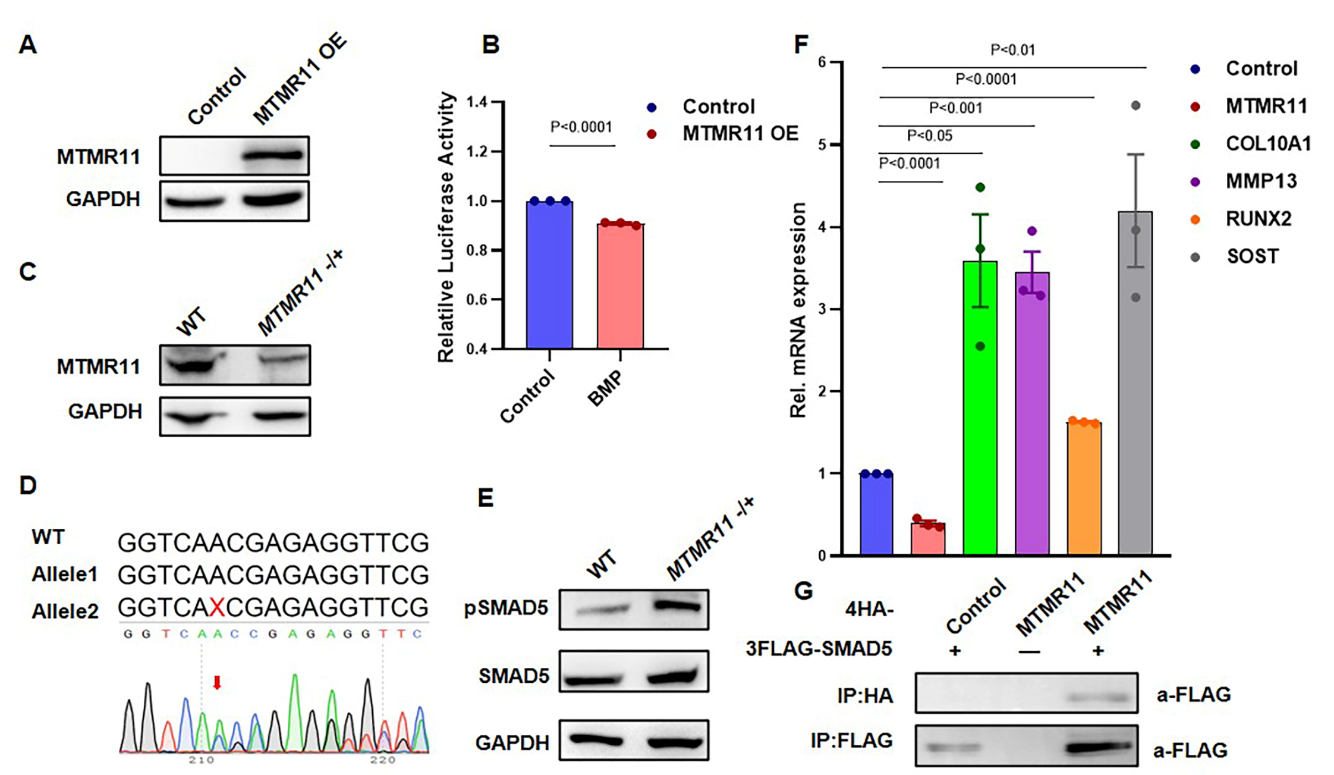


**Fig.S2 MTMR11 interacted with SMAD5 and inhibited the BMP signaling pathway.** (A) Overexpression (OE) of MTMR11 protein; empty vector (EV) was used as a control. (B) Overexpression (OE) of *MTMR11* inhibits the BMP signaling pathway (two-tailed t test, n=3), and empty vector (EV) was used as a control. (C-D) Expression of MTMR11 protein and sequencing of the *MTMR11* gene in the heterozygous *MTMR11* KO (*MTMR11-/+*) cell line. Red X and red arrows represent the location of missing deoxyribonucleic acid bases. (E) Upregulation of phosphorylated-SMAD5 (pSMAD5) in the heterozygous *MTMR11* KO cell line. (F) Higher expression of the BMP pathway marker genes *COL10A1*, *SOST*, *MMP13*, and *RUNX2* in the *MTMR11* KO cell line (two-tailed t test, n=3), empty vector (EV) as a control. (G) Coimmunoprecipitation (Co-IP) showed that MTMR11 could interact with SMAD5 in the HEK293T cell line, with empty vector (EV) as a control, SMAD5 with a 3XFLAG tag, and MTMR11 with a 4XHA tag.


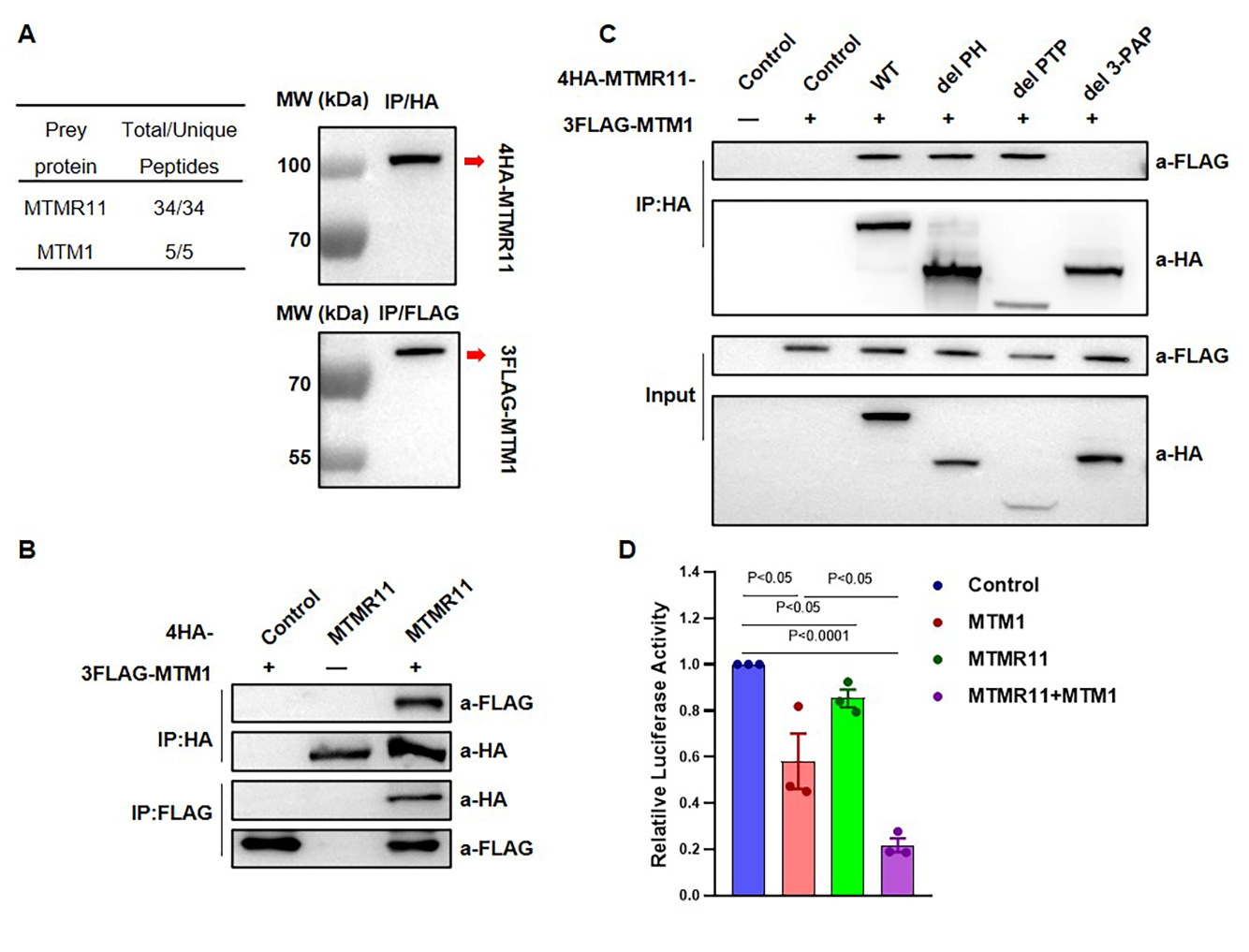


**Fig. S3 MTMR11 interacted with MTM1 to enhance MTM1's inhibition of the BMP pathway.** (A) MS analysis showed an interaction between MTM1 and MTMR11. MTMR11 with 4XHA tag, MTM1 with 3XFLAG tag. (B) Co-IP showed the interaction of MTMR11 and MTM1 *in vitro*. (C) Co-IP demonstrated that MTMR11 without the 3-PAP domain lost its interaction with MTM1 (blue arrow). Del PH, del PTP, and del 3-PAP represent the deletion of the PH, PTP, and 3-PAP domains, respectively. Empty vector was used as a control. (D) Luciferase assay showed the inhibitory role of *MTM1* on the BMP signaling pathway, which was further exaggerated by cotransfection with *MTMR11*. Empty vector as control, two tailed t test, n=3.


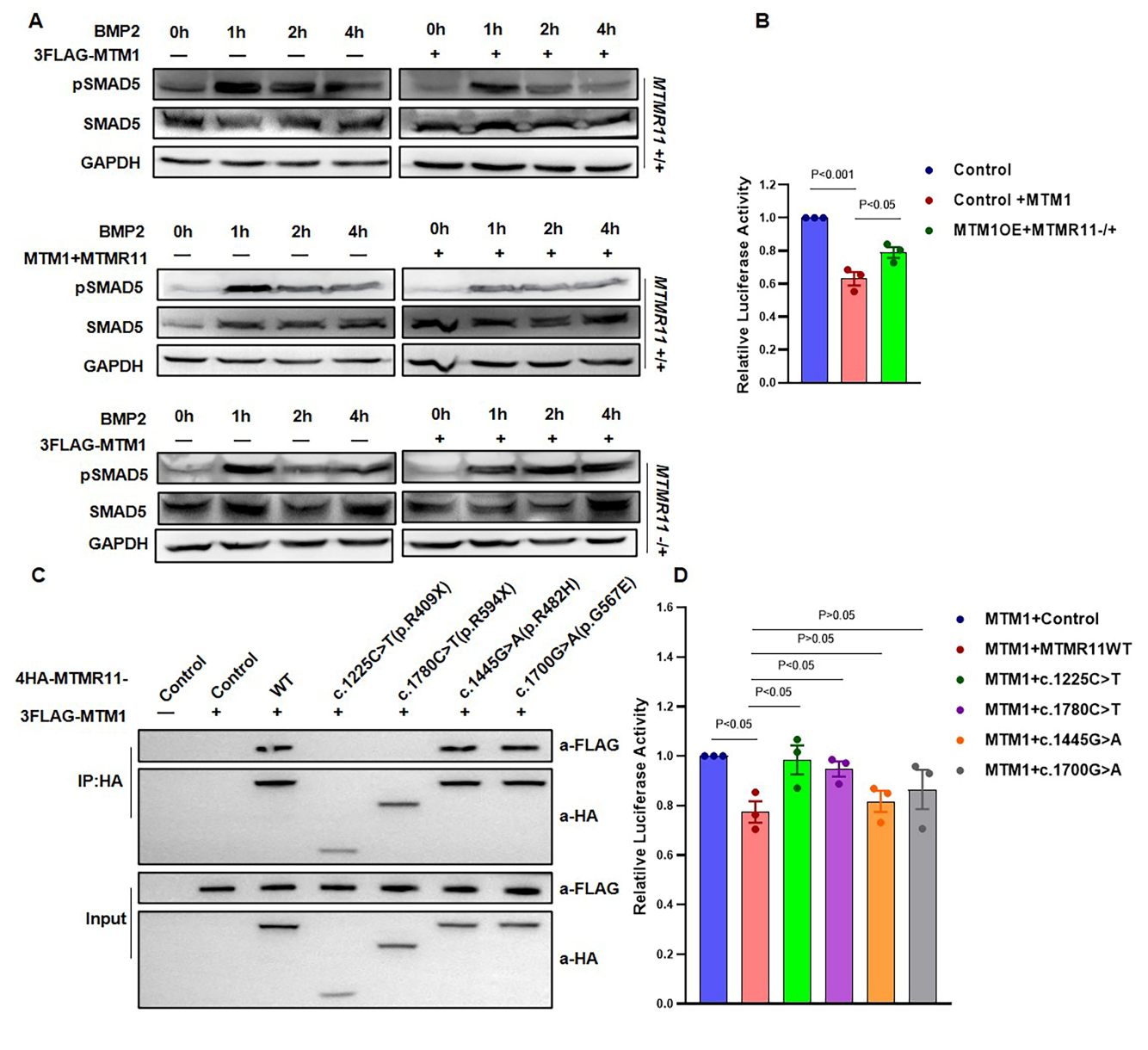


**Fig. S4 *MTMR11* acted as an essential co-factor to enhance MTM1 dephosphorylation of pSMAD5**

(A) When levels of pSMAD5 rose with external BMP2 treatment (h: hours), overexpression of *MTM1* and *MTMR11* downregulated the expression of pSMAD5 in *MTMR11*+/+ cells, which could be partly recovered in *MTMR11*-/+ cells. (B) When MTM1 was overexpressed, BMP activity was significantly decreased (p<0.001) in *MTMR11*+/+ cells but could be partially recovered (p<0.05) in *MTMR11*-/+ cells. (C) Co-IP results indicate that the interaction between MTM1 and MTMR11 variants p. R409X and p. R594X vanished, but the MTM1 interactions with variant p. R482H or variant p. G567E are similar to wild-type MTMR11. (D) The luciferase assay in *MTMR11*-/+ cells showed that compared to the empty vector control, the BMP activity was significantly decreased (p<0.05) when transfected with *MTM1* only. The decreased BMP activity was restored with cotransfection of MTM1 and MTMR11 variant p. R409X (p<0.05) or p.R594X (p<0.05), but no recovery when cotransfected with MTM1 and the MTMR11 variant p. R482H (p>0.05) or variant p. G567E (p>0.05) (two tailed t test, n=3).


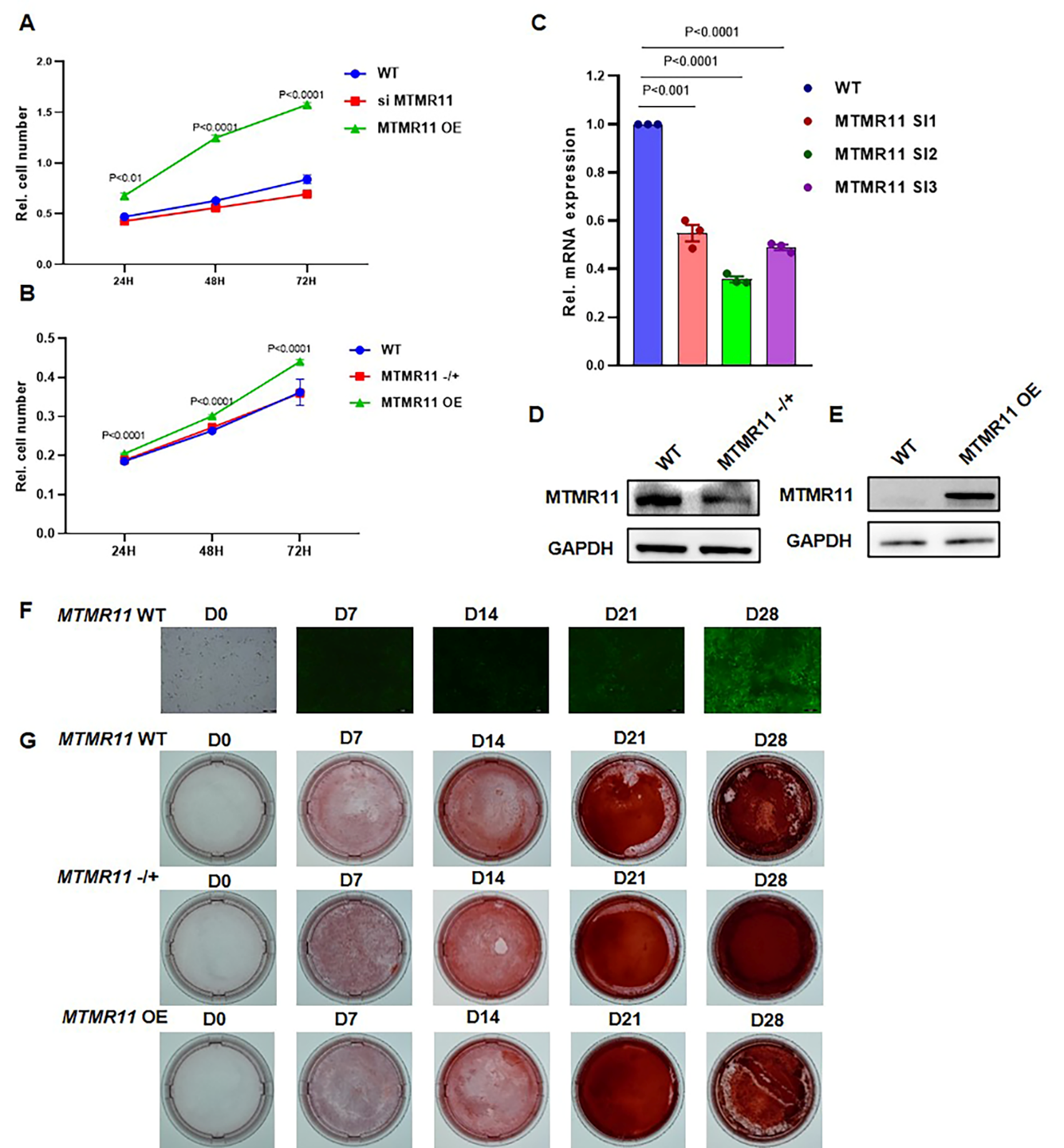


**Fig. S5 *MTMR11* accelerated proliferation and inhibited osteoblast differentiation of IDG-SW3 cells.**

(A) Proliferation of U-2OS cells was significantly increased with the overexpression of *MTMR11* but showed no significant difference from the wild-type controls when *MTMR11* was knocked down by si *MTMR11*. (B) Proliferation of IDG-SW3 cells was significantly increased with the overexpression of *MTMR11* but showed no significant difference from the wild-type controls when *MTMR11* was knocked out (*MTMR11-/+*). (C) *MTMR11* was knocked down to less than half of the wild control after being individually transfected with three pairs of siRNAs (si1, si2, si3). Empty vector as control, two tailed t test, n=3. (D) Western blotting showed that the MTMR11 protein level in IDG-SW3 cells of *MTMR11-/+* is less than half that of the wild type. (E) Western blotting detected MTMR11 OE in IDG-SW3 cells. (F) Expression level of the Dmp1-GFP fusion protein on differentiation Days 0/7/14/21/28. (G) Alizarin red S staining on differentiation Day 0/7/14/21/28 *in* *MTMR11* WT, *MTMR11*-/+, and *MTMR11* OE cell lines: Alizarin red S staining density on Day 28 was markedly enhanced in the MTMR11-/+ cell line (Fig. 5G, middle line), and the staining density of MTMR11 OE cells (Fig. 5G, third line) was decreased compared to that of MTMR11 WT cells (Fig. 5G, first line).


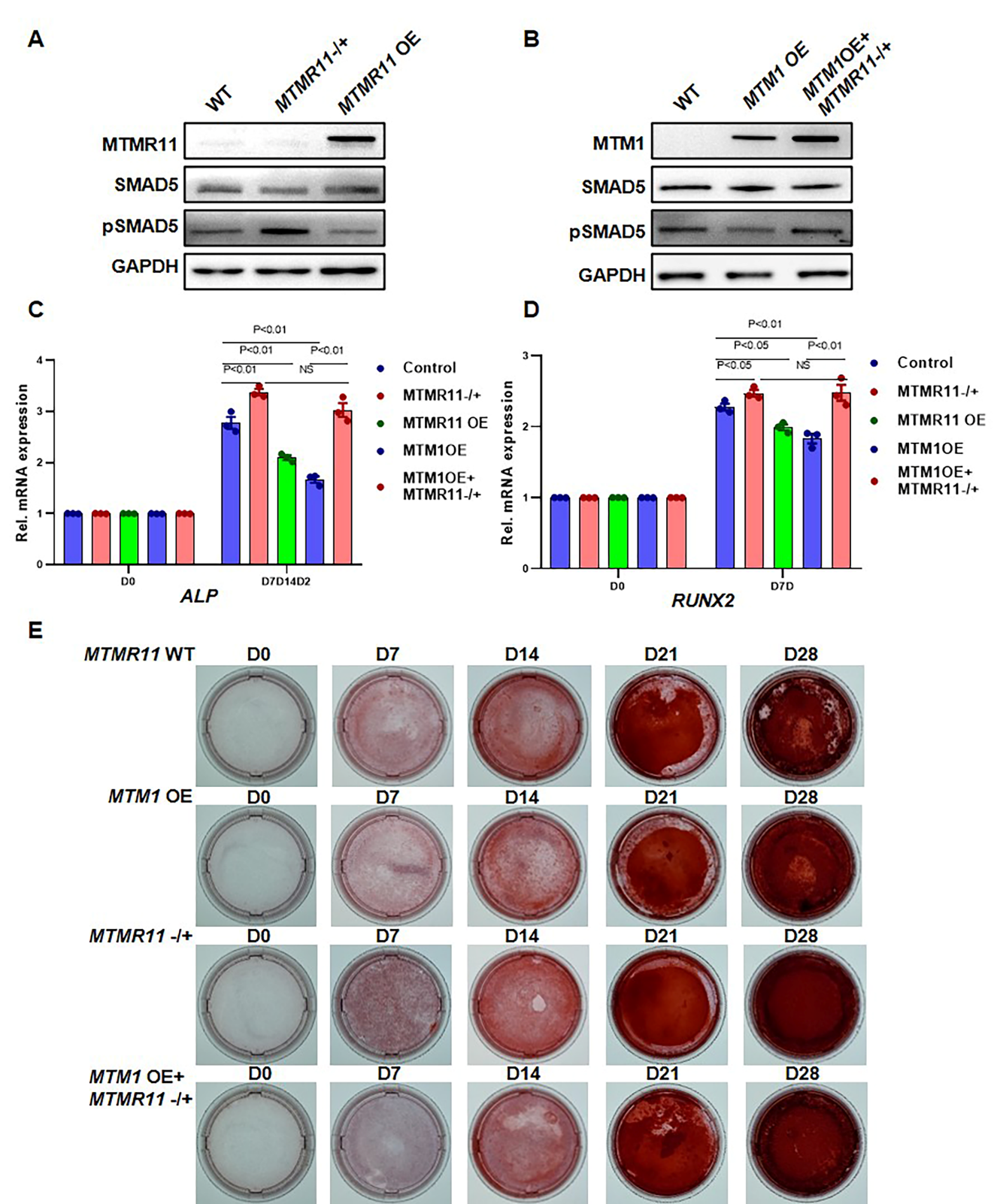


**Fig. S6 MTM1's inhibition of osteoblast differentiation in IDG-SW3 cells was compromised by knockout of *MTMR11*.**

(A, B) Expression of pSMAD5 was downregulated by overexpression of *MTM1* and *MTMR11* in *MTMR11*+/+ IDG-SW3 cells but partly recovered in *MTMR11*-/+ IDG-SW3 cells. (C, D) Expression of *ALP* and *RUNX2* was downregulated by overexpression of *MTM1* and *MTMR11* on differentiation Day 0/28 in IDG-SW3 cell lines and partly recovered in *MTM1* OE + *MTMR11*-/+ cells. (E) On differentiation Day 28, significantly lighter Alizarin red S staining was observed in *MTMR11* WT cells when transfected with MTM1, while no significant stain difference was observed in *MTMR11*-/+ cells when transfected with MTM1.
